# Improved Antileishmanial Activity of Dppz through Complexation with Antimony(III) and Bismuth(III): Investigation of the Role of the Metal

**DOI:** 10.3390/molecules171112622

**Published:** 2012-10-25

**Authors:** Edgar H. Lizarazo-Jaimes, Rubens L. Monte-Neto, Priscila G. Reis, Nelson G. Fernandes, Nivaldo L. Speziali, Maria N. Melo, Frédéric Frézard, Cynthia Demicheli

**Affiliations:** 1 Department of Chemistry, Institute of Exact Sciences, Federal University of Minas Gerais (UFMG), Av. Antônio Carlos 6627, 31270-901, Belo Horizonte, MG, Brazil; 2 Department of Physiology and Biophysics, Institute of Biological Sciences, Federal University of Minas Gerais (UFMG), Av. Antônio Carlos 6627, 31270-901, Belo Horizonte, MG, Brazil; 3 Department of Physics, Institute of Exact Sciences, Federal University of Minas Gerais (UFMG), Av. Antônio Carlos 6627, 31270-901, Belo Horizonte, MG, Brazil; 4 Department of Parasitology, Institute of Biological Sciences, Federal University of Minas Gerais (UFMG), Av. Antônio Carlos 6627, 31270-901, Belo Horizonte, MG, Brazil

**Keywords:** crystal structure, dipyrido [3,2-a:2',3'-c] phenazine, antimony, bismuth, *Leishmania*, drug resistance

## Abstract

Two novel trivalent antimony(III) and bismuth(III) complexes with the nitrogen-donor heterocyclic ligand dipyrido[3,2-a:2',3'-c]phenazine (dppz) were synthesized and characterized as [Sb(dppz)Cl_3_]∙H_2_O∙CH_3_OH and [Bi(dppz)Cl_3_]. The crystal structure of Sb(III) complex was determined by X-ray crystallography. These complexes were evaluated for their activity against the promastigote form of Sb(III)-sensitive and -resistant *Leishmania infantum chagasi* and *Leishmania amazonensis* strains. Both complexes were more effective than dppz alone in inhibiting the growth of *Leishmania* promastigotes and were at least 77 and 2,400 times more active than potassium antimonyl tartrate in Sb(III)-sensitive and -resistant *Leishmania*, respectively. The cytotoxicity of dppz and its complexes against mouse peritoneal macrophages occurred at dppz concentrations at least 6-fold greater than those found to be active against *Leishmania* promastigotes.To investigate the role of the metal in the improved antileishmanial activity of dppz, the activity of the Sb(III) complex was compared between the Sb-resistant mutants and their respective parental sensitive strains. The lack of cross-resistance to the Sb(III)-dppz complex together with the much lower activity of antimonyl tartrate, SbCl_3_ and BiCl_3_ strongly support the model that the metal is not active by itself but improves the activity of dppz through complexation.

## 1. Introduction

Pentavalent antimonials such as sodium stibogluconate and meglumine antimonate, have been used in the treatment of all forms of leishmaniasis for more than half a century [1,2]. Although the mechanism of action of pentavalent antimonial is not fully understood, it is generally accepted that the active form of the metal is the reduced form Sb(III) [3,4].

A major problem in antimonial chemotherapy is the emergence of clinical resistance against pentavalent antimony drugs that has reached epidemic proportions in parts of India [5]. The ATP binding cassette (ABC) protein MRPA plays a major role in metal resistance in *Leishmania* parasites [6] and its localization in intracellular vesicle membranes suggests that it sequesters Sb(III)-thiol complexes into these vesicles [7].

Other mechanisms such as a diminished biological reduction of Sb(V) to Sb(III) [8], the loss of an aquaglyceroporin (AQP1) allele or its down regulation [9] and hypoxic conditions [10] have been reported to cause an increase in resistance to pentavalent antimonials. In this context, there is a great need for new safe and effective drugs that do not exhibit cross-resistance with conventional antimonial drugs. 

Antimony(III) complexes have attracted special interest as potential antineoplastic agents since 1990, when Silvestru *et al.* [11] reported for the first time the anti-tumor activity of Sb(III) complexes. It was suggested that the mode of action of trivalent antimonial compounds involves some pathways similar to apoptosis, as DNA fragmentation [12,13], which is preceded by an increase in reactive oxygen species caused by alterations of the redox potential [14]. As an antileishmanial agent, Sb(III) inhibits and forms a complex with the enzyme trypanothione reductase which acts by recycling trypanotione disulphides in trypanothione, the major antioxidant thiol in *Leishmania*, important to maintain their intracellular redox balance [15,16].

On the other hand, bismuth compounds have been used to treat infections caused by *Helicobacter Pylori* bacteria and duodenal ulcers [17], as well as radio-therapeutic agents for cancer treatment [18]. Despite the close periodic table relationship of antimony and bismuth, there are only few reports in the literature of bismuth-based drugs being developed and evaluated as antileishmanial agents [19]. On the other hand, new bismuth complexes with DNA affinity and activity against cancer cell were reported [20,21].

Metal complexes with polypyridyl ligands, have been extensively studied by a number of research groups over the last year due to potential application in photodynamic therapy [22,23] and probes for biological molecules [24,25].

Recently, complexes of dipyrido[3,2-a:2',3'-c]phenazine (dppz) with gold (Au), copper (Cu) and vanadium (V) have shown remarkable antileishmanial or antitrypanosomal activities [26,27], however, the possible synergism between the metal and the ligand was not fully investigated. On the other hand, there are few reports in the literature referring to the structural characterization of Sb(III) and Bi(III) complexes with polypyridyl ligands [28,29], but no report on their pharmacological activities.

In this work, the synthesis, the structural and physicochemical characterization of the polypyridyl ligand dppz coordinated to Sb(III) and Bi(III) are reported. The activities of dppz and its resulting complexes against both Sb(III)-sensitive and -resistant *Leishmania infantum chagasi* and *Leishmania amazonensis* promastigotes and their cytotoxicities towards peritoneal macrophages are described. The present paper reports for the first time a new experimental approach, based on tests against metal-resistant mutants and their respective parental sensitive strains, to get insight into the role of the metal in the cytotoxicity of a metal complex.

## 2. Results and Discussion

Antimony and bismuth complexes [Sb(dppz)Cl_3_] (**1**) and [Bi(dppz)Cl_3_] (**2**) were obtained in good yields by refluxing equimolar amounts of SbCl_3_ or BiCl_3_ with dppz in methanolic solutions. The complexes **1** and **2** prepared during the course of this investigation are an air-stable crystal and powder, respectively, which were characterized by spectroscopic methods including FT-IR, NMR, elemental analysis and also (in the case of complex **1**) by X-ray crystallography. In the case of complex **2**, the crystals obtained did not show sufficient quality for X-ray analysis. In FT-IR spectra of complexes **1** and **2**, bands were observed for C=N and C=C bonds stretching in aromatics in the 1640-1400 cm^−1^ range. The dppz bands at around 1632 and 1616 cm^−1^ are shifted considerably towards lower frequency—1616 and 1602 cm^−1^ for [Sb(dppz)Cl_3_] and 1616 and 1598 cm^−1^ for [Bi(dppz)Cl_3_]—confirming the coordination of N atoms to Sb and Bi. Similar results for the spectroscopic data of dppz complexes with Au and Cu have been reported previously [26]. The corresponding ^1^H-NMR spectra showed only one set of signals, shifted downfield in both complexes with respect to the free ligand (Table 1). ^1^H-NMR spectra of [Sb(dppz)Cl_3_] and [Bi(dppz)Cl_3_] complexes in DMSO-*d_6_* exhibited the presence of the polypyridine ligand aromatic protons between δ 9.68 and 8.05 ppm. 

**Table 1:** Selected ^1^H-NMR and IR data for free ligand and complexes **1** and **2**.

Compound	^1^H-NMR data (ppm)					References
	Hc	Ha	Hd	He	Hb	
Dppz	9.55	9.23	8.41	8.07	7.95	Navarro *et al*. (2006) [26]
*Sb(dppz)Cl_3_*	9.68	9.31	8.39	8.18	8.11	This work
*Bi(dppz)Cl_3_*	9.54	9.39	8.32	8.07	8.05	

**Figure 1:**
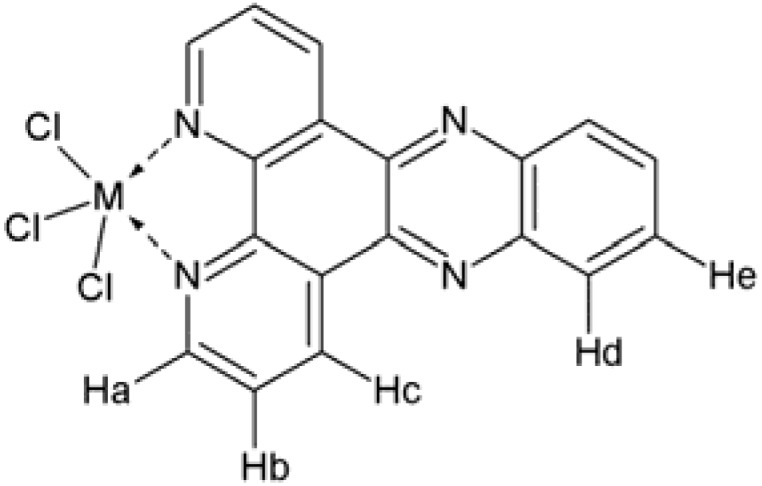
Molecular structure of [M(dppz)Cl_3_], with M= Sb or Bi.

The *Hc* proton of dppz and both complexes showed resonances at very low field because they are also highly deshielded due to the anisotropic effect provided by the nitrogen atom of the pyrazine ring (Figure 1). *Ha* protons in proximity of the coordinating nitrogen atoms of dppz ligand experienced a downfield shift by about δ 0.08-0.16 in complexes **1** and **2**, respectively, indicating the involvement of the dppz nitrogens in coordination. The unequivocal proton NMR assignments were made by concerted analysis of 1D ^1^H-, 1D ^13^C-, 2D ^1^H ^1^H COSY and 2D ^1^H ^13^C-HMQC.

**Figure 2:**
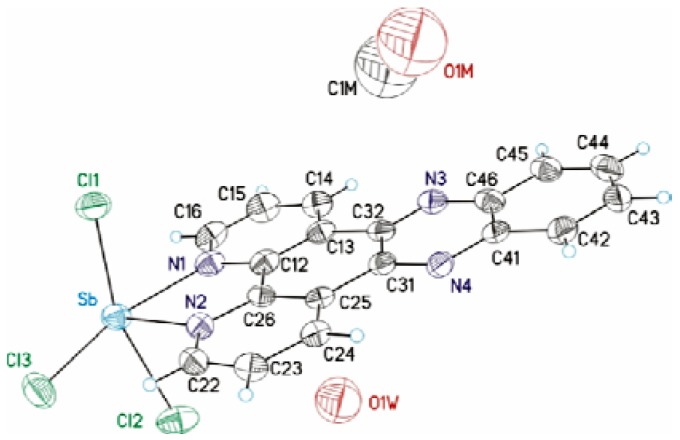
Molecular structure of [Sb(dppz)Cl_3_].

For both complexes, satisfactory elemental analyses of C, H and N were obtained. In complex **1** crystallization methanol and water molecules are present, as confirmed by its thermogravimetric curves which showed a weight loss of 8.26% (calc. 8.92%), nevertheless complex **2** did not have crystallization molecules. The asymmetric unit of complex **1** is shown in Figure 2. Crystal data and structure refinement parameter are given in Table 2 and the selected bonds length and angles are given in Table 3.

**Table 2:** Crystal data and structure refinement parameter for complex **1**.

	Sb(dppz)Cl_3_
Empirical formula	C_19_H_10_Cl_3_ N_4_O_1.25_Sb
Formula weight	542.41
Temperature (K)	293(2)
Crystal system	Monoclinic
Space group	P2(1)/c
a (Å)	10.3244(2)
b (Å)	13.2760(3)
c (Å)	14.5753(3)
*α* (^o^)	90
*β* (^o^)	92.0930(19)
*γ* (^o^)	90
*V* (Å^3^)	1994.44(7)
*Z*	4
*F* (000)	1056
D_calc_ (mg/m^3^)	1.805
Crystal dimensions mm^3^	0.2 × 0.2 × 0.3
*θ* Range (^o^)	4.29-62.65
Reflections collected	9035
Independent reflection	3138
*R_int_*	0.0355
Maximum/minimum transmission	1.000 / 0.30303
Data/restraints/parameters	3138 / 0 / 247
Goodness-of-fit on *F^2^*	1.060
Final *R* indices	*R1* = 0.0352, w*R2* = 0.0826
[I > 2σ(I)]	2478
*R* indices (all data)	*R1* = 0.0522, w*R2* = 0.0886
Largest difference in peak/hole (e·A^−3^)	0.761 and −0.560

**Table 3:** Selected bond length (Å) and angles (°) for complex **1**.

Sb-N(2)	2.245(4)	N(2)-Sb-N(1)	71.44(14)
Sb-N(1)	2.345(4)	N(1)-Sb-Cl(3)	159.07(10)
Sb-Cl(3)	2.4992(15)	N(2)-Sb-Cl(3)	87.73(11)
Sb-Cl(1)	2.5126(13)	N(2)-Sb-Cl(1)	82.14(10)
Sb-Cl(2)	2.6348(14)	N(1)-Sb-Cl(1)	84.23(10)
N(1)-C(16)	1.334(6)	Cl(3)-Sb-Cl(1)	95.22(5)
N(1)-C(12)	1.351(6)	N(2)-Sb-Cl(2)	80.25(10)
N(2)-C(22)	1.336(6)	N(1)-Sb-Cl(2)	82.97(10)
N(2)-C(26)	1.368(6)	Cl(3)-Sb-Cl(2)	91.68(5)
		Cl(1)-Sb-Cl(2)	160.80(5)

The geometry around the metal center is distorted square pyramidal (SP), with N(2) as the apex and Cl(1), Cl(2), Cl(3), N(1) occupying the equatorial plane. The four equatorial bonds are long (means 2,498 Å), while the apical donor atom form the strongest bond (Sb(1)-N(2) 2.245(4) Å). The sum of equatorial angles is 354° (Cl(3)-Sb-Cl(1) 95.22(5)°, Cl(3)-Sb-Cl(2) 91.68(5)°, N(1)-Sb(1)-Cl(1) 84.23(10)°, N(1)-Sb(1)-Cl(2) 82.97(10)°), which suggest some distortion from an ideal square pyramidal [30]. As shown in Figure 2, the asymmetrical unit in the crystal contains one molecule of methanol and one of water besides the [Sb(dppz)Cl_3_] molecule.

**Figure 3:**
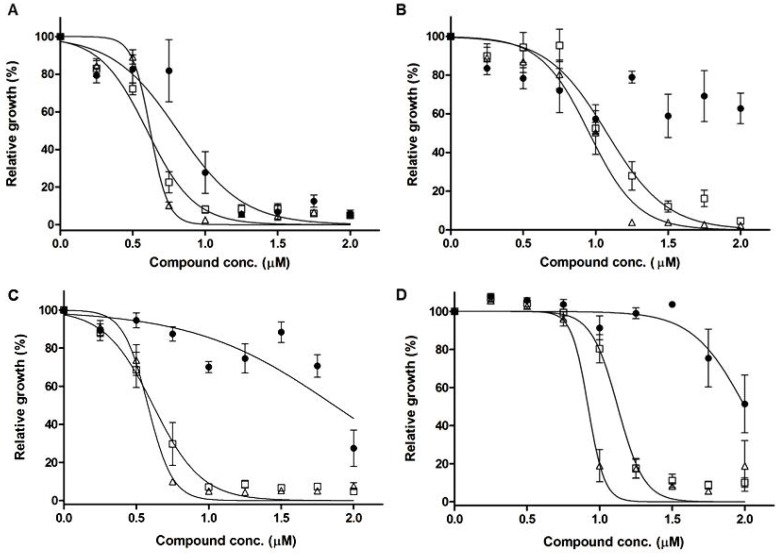
Dppz-derived Sb(III) and Bi(III) complexes sensitivity profiles of antimony-sensitive and -resistant *L. chagasi* and *L. amazonensis* promastigotes. (**A**) *L. chagasi* BH400 wild-type; (**B**) *L. amazonensis* BA199 wild-type; (**C**) *L. chagasi* BH400Sb2700.2 (Sb-resistant); (**D**) *L. amazonensis* BA199Sb2700.2 (Sb-resistant). (•) dppz; (□) dppz/Bi; (∆) dppz/Sb. The *Leishmania* promastigotes were cultivated in 24-well cell culture plates containing 2 mL of α-MEM medium for 72 h at 25 °C. The resistant lines were previously selected *in vitro* by step wise drug selection. All data represent average of, at least, three independent experiments.

Both complexes **1** and **2 **were found to be highly active against *Leishmania* promastigote forms (Figure 3). The IC_50_ for complexes **1** and **2** in the wild-type (WT) strains were similar, corresponding to ~0.6 and ~1 μM for *L. infantum chagasi* and *L. amazonensis*, respectively (Table 4).

Strikingly, the IC_50_ values for dppz complexes were similar when comparing the Sb-resistant mutants with their respective parental sensitive WT strains (Table 4). This is in contrast with the trivalent potassium antimonyl tartrate that was much less active against Sb-resistant mutants (Table 4).

**Table 4:** Inhibitory concentrations of dppz and its Sb(III) and Bi(III) complexes towards antimony-sensitive and -resistant New World *Leishmania* species.

Strain	IC_50_ (μM) ± SEM (CI 95%)					
	Dppz	dppzBiCl_3_	dppzSbCl_3_	TA *	SbCl_3_	BiCl_3_
*L. infantum chagasi* (WT)	0.81 ± 0.04	0.59 ± 0.01	0.62 ± 0.01	100 ± 3	341 ± 2	563 ± 2
	(0.71–0.9)	(0.56–0.63)	(0.59–0.65)			
*L. infantum chagasi* (SbR)	1.86 ± 0.08	0.61 ± 0.02	0.57 ± 0.01	>2,700	462 ± 3	>500
	(1.69–2.03)	(0.56–0.66)	(0.55–0.60)			
*L. amazonensis* (WT)	>2	1.07 ± 0.03	0.95 ± 0.02	83 ± 1	362 ± 2	621 ± 2
		(1.01–1.14)	(0.89–1.01)			
*L. amazonensis* (SbR)	2.00 ± 0.05	1.12 ± 0.01	0.92 ± 0.02	>2,700	1503 ± 2	>500
	(1.89–2.10)	(1.08–1.16)	(0.86-0.97)			

* TA: potassium antimonyl tartrate as Sb(III) source; SEM:standard error of the mean; CI: confidence interval; SbR (Sb-resistant); The IC_50_ values were calculated by non-linear regression.

When tested alone, dppz showed intrinsic activities against *Leishmania*, with IC_50_ varying from 0.8 to ~2 μM for both Sb-sensitive or -resistant mutants of the two species studied (Table 4). Interestingly, complexes **1** and **2**exhibited significantly higher antileishmanial activity than dppz itself, indicating an additional role of the metal (Sb(III) and Bi(III)) in the antileishmanial activity of the complexes (Figure 3 and Table 4). Since complexes **1** and **2** were at least 80-fold more active against WT *Leishmania* strains than trivalent potassium antimonyl tartrate, BiCl_3_ and SbCl_3_ (Table 4), one can infer that the metal may not be active by itself and that the higher activity of the complexes compared to dppz may be related to the improved activity of dppz through metal complexation. The lower IC_50_ value of antimonyl tartrate compared to SbCl_3_ in WT *L. amazonensis* also illustrates that complexation of a cytotoxic metal ion may enhance its activity, presumably through improved delivery to the target. In the case of antimonyl tartrate, much greater IC_50_ values were found in Sb-resistant mutants compared to the respective WT parental cells, demonstrating clearly that the metal ion contributes to the cytotoxicity of this Sb(III) complex. This is in contrast with the Sb(III)-dppz complex that was equally active against Sb-resistant and -sensitive *Leishmania* strains. Thus, the latter observation further supports the model that the metal is not directly responsible for the antileshmanial activity of Sb(III)-dppz complex. As shown in Table 5, the cytotoxicity of dppz and its complexes against mouse peritoneal macrophages occurred at dppz concentrations at least 6-fold greater than those found to be active against *Leishmania* promastigotes, indicating the selectivity of the complexes towards *Leishmania* parasites.

**Table 5:** Cytotoxicity of dppz and its Sb(III) and Bi(III) complexes against mouse peritoneal macrophages.

	Dppz	[Bi(dppz)Cl_3_]	[Sb(dppz)Cl_3_]
CC_50_ (μM) *	12.5	4.8	7.0
SI ^#^	15.4	8.1	11.3

* CC_50_: concentration of dppz which is cytotoxic against 50% of macrophages; ^#^ SI: selective index, calculated as the ratio between CC_50_ in murine macrophages and IC_50_ in *L. infantum chagasi* (WT).

The *L. infantum chagasi* is the etiological agent for visceral leishmaniasis [31] while *L. amazonensis* is related to the cutaneous form of the disease in the New World [32]. Since these two species are causative agents of different clinical manifestations, they might also differ metabolically which reflect in their distinct drug sensitivity. This observation highlights the importance to use different *Leishmania* species for drug prospecting screening [33]. In addition to the high antileishmanial activity of **1** and **2**, both complexes can bypass the Sb-resistance of *Leishmania* in two different species of Sb-resistant *Leishmania* mutants (Table 4). The Sb-resistant selected lines of *L. infantum chagasi* BH400 Sb2700.2 and *L. amazonensis* BA199 Sb2700.2 were able to grow in the presence of Sb concentrations greater than 2700 μM, but failed to grow up in the presence of ≤1.12 μM of **1** and/or **2** (Table 4). Thus, this data taken altogether indicates that complexes **1** and **2** are very promising drug candidates for the treatment of Sb-resistant leishmaniasis.

An important physical characteristic that affects the bioavailability of a compound is its lipophilicity. Therefore a relatively optimal lipophilicity should be critical for a good drug candidate.

The lipophilicity of the complexes **1** and **2** was determined by measuring their octanol/water partition coefficients (log P). The log P values of the free dppz ligand was calculated using the online program ALOGSP 2.1. The log P values for dppz and complexes **1** and **2** are respectively 2.77 ± 0.76; 0.94 ± 0.24 and 0.98 ± 0.17. More positive log P values correspond to more lipophilic compounds, whereas more negative log P values correspond to more hydrophilic ones. This data indicates that complexation with Sb(III) and Bi(III) makes dppz less lipophilic. Interestingly, the antileishmanial activity profile follows the hydrophilicity profile of the compound, *i.e.*, the more hydrophilic, the more active.

A possible explanation for the leishmanicidal activity of dppz complexes is their interaction with parasite DNA through intercalation, a mechanism recently suggested by Navarro and co-workers [34]. They described both Cu(II) and Au(III) complexes with dppz showing a potent leishmanicidal activity against both promastigotes of *Leishmania* (*Leishmania*) *mexicana and Leishmania* (*Viannia*) *braziliensis* [34]. Although the best-known type of interaction of the polypyridyl complexes with DNA is the intercalation, highly reactive metal complexes containing reactive Cl could form the covalent bonds with DNA, as reported for [Ru(tpy)(pap)(CH_3_CN)](ClO_4_)_2_and [Ru(tpy)(dppz)(CH_3_CN)]^2+^[35,36]. In future studies, the interaction of Sb(III) and Bi(III) dppz complexes with DNA should be investigated, as a possible mechanism of cytotoxicity. In our work the differences in observed cytotoxicities of the complexes compared to dppz could be attributed to the changes in dppz lipophilicity following the coordination by SbCl_3_ and BiCl_3_. The metal complexation to dppz probably leads to an increase of dppz bioavailability.

## 3. Experimental

### 3.1. General

Commercially available methanol was dried before use. All the preparations were done under anhydrous conditions. dppz was synthesized by a condensation of 1,10-phenanthroline-5,6-dione and *ortho*-phenylenediamine according to the procedure described by Liang [37]. Sb(III) chloride (SbCl_3_) and Bi(III) chloride (BiCl_3_), from Sigma-Aldrich (St. Louis, MO, USA) were used without further purification. Elemental analyses for C, H and N were carried out with a Perkin Elmer PE-2400 instrument. IR spectra in the 4,000–400 cm^−1^ region were obtained for KBr pellets, with a Perkin Elmer RX-83303. The absorption spectra were recorded on a Varian Cary 100 *UV-Visible* Spectrophotometer.

NMR spectra were recorded at 400 MHz using Bruker DPX-400 spectrometer. ^1^H- and ^13^C-NMR chemical shifts were measured relative to tetramethylsilane (TMS) and dimethyl sulfoxide-d_6_ (DMSO-*d*_6_) as the solvent. Intensity data for the X-ray were collected with Xcalibru Atlas Gemini ultra. Crystallographic data and experimental details of the structure determinations are listed in Table 1. 

### 3.2. Synthesis of the Complexes [Sb(dppz)Cl_3_] and [Bi(dppz)Cl_3_]

#### 3.2.1. Synthesis of [Sb(dppz)Cl_3_] (**1**)

A solution of dipyrido[3,2-a:2',3'-c]phenazine (0.05 g, 0.18 mmol) in dry methanol (26 mL) was added slowly to a freshly prepared antimony trichloride (0.041 g SbCl_3_, 0.18 mmol in 10 mL of dry methanol) solution at room temperature. The reaction mixture was stirred for 6 hours at 40 °C. The resulting clear solution was kept in the darkness at room temperature to give yellow crystals. Yield: 82.9% (0.92 g). IR (KBr, cm^−1^): 1614, 1578 *ν*(C=C, C=N); 1,138, 818, 724 δ(Csp^2^-H). ^1^H-NMR (DMSO-*d*_6_ 400 MHz): δ 9.68 (dd, Hc); 9.31 (dd, Ha); 8.39 (q, Hd); 8.21–8.08 (m, He and Hb). Thermogravimetric and crystal data indicated the presence of one crystallization molecule of water and methanol. Elemental analysis for C_19_H_16_Cl_3_N_4_O_2_Sb, calc. (found): C% 40.70 (41.21); H% 2.86 (2.73); N% 10.00 (10.25).

#### 3.2.2. Synthesis of [Bi(dppz)Cl_3_] (**2**)

A solution of dipyrido[3,2-a:2',3'-c]phenazine (0.07 g, 0.25 mmol) in dry methanol (26 mL) was added slowly to a freshly prepared antimony trichloride (0.079 g BiCl_3_, 0.25 mmol in 10 mL of dry methanol) solution at room temperature. The reaction mixture was stirred for 3 h at 40 °C and the resulting precipitatate was filtered and dried. Yield: 82.9% (0.123 g). IR (KBr, cm^−1^): 1,614, 1,574 *ν*(C=C, C=N); 1,140, 816, 730 δ(Csp^2^-H). ^1^H-NMR (DMSO-*d*_6_ 400 MHz): δ 9.54 (dd, Hc); 9.39 (dd, Ha); 8.32 (q, Hd); 8.04–8.08 (m, He and Hb). Elemental analysis for C_18_H_12_Cl_3_N_4_Bi, calc. (found): C% 36.15 (36.21); H% 1.67 (1.63); N% 9.37 (9.19).

#### 3.2.3. Stability of the Complexes

The stability of the complexes was checked by UV absorption spectroscopy. For this purpose, UV absorption spectra were registered at different time intervals (from 0 to 4 h) for dppz and complexes **1** and **2** at 10 µmol·L^−1^ in DMSO, DMSO-containing water and octanol. The spectra of the complexes were different from that of dppz and did not show significant changes as a function of time.

### 3.3. X-ray Crystallography

Single crystal X-ray diffraction data were obtained at room temperature on a Xcalibur Atlas Gemini Ultra diffractometer, using graphite monochromated MoKα radiation (λ = 0.71069 Å). Final unit cell parameters and the integration of the collected reflections were performed using the CRYSALISPRO software (Version 1.171.33.55 release 05-01-2010 CrysAlis171.NET). The structure solutions and full-matrix least-squares refinements based on *F*^2^ were performed with the SHELXS-97 and SHELXL-97 program package [38]. All atoms except hydrogen were refined anisotropically. Although many hydrogen atoms could be identified in a Fourier difference map all of them were geometrically added to the structure and then refined by the riding model in the final stages. Details of data collection and structure refinement are given in Table 2. Selected distances and angles are given in Table 3. The crystallographic data were deposited at Cambridge Crystallographic Data Center on CCDC 874746.

### 3.4. Antileishmanial and Cytotoxic Activities

#### 3.4.1. Parasite Culture

*Leishmania* (*Leishmania*) *amazonensis* (strain MHOM/BR/1989/BA199) and *Leishmania* (*Leishmania*)*infantum chagasi* (strain MCAN/BR/2002/BH400) promastigotes were maintained in minimum essential culture medium (α-MEM) (Gibco, Invitrogen, Grand Island, NY, USA) supplemented with 10% (v/v) heat inactivated fetal calf serum (Cultilab, Campinas, SP, Brazil), 100 mg/mL kanamycin, 50 mg/mL ampicillin, 2 mM L-glutamine, 5 mg/mL hemin, 5 mM biopterin, (Sigma-Aldrich), pH 7.0 and incubated at 25 °C. *L. amazonensis* and *L. infantum chagasi* were selected for Sb(III) resistance as previously decribed [39,40]. The Sb(III)-resistant mutants *L. amazonensis* BA199Sb2700.2 and *L. infantum chagasi* BH400Sb2700.2 were selected in 25 cm^2^ flasks containing 5 mL of α-MEM in the presence of increasing Sb(III) concentrations up to 2,700 μM.

#### 3.4.2. Antileishmanial Activity Assay

Complexes **1** and **2** were evaluated *in vitro* for their activity against both Sb(III)-sensitive and -resistant *Leishmania* parasites. Log-phase *L. amazonensis* and *L. infantum chagasi* promastigotes (1 × 10^6^ parasites/mL) were seeded in 24-wells cell culture plates with 1.5 mL of α-MEM, incubated under shaking at 25 °C during 72 h in the presence of several concentrations of complex **1** or **2**. Controls were performed using cultures in the presence of potassium antimonyl tartrate, SbCl_3_, BiCl_3_ and dppz itself. Non-treated parasites were established for growth comparison. Stock solutions of dppz, its complexes, SbCl_3_ and BiCl_3_ were dissolved in DMSO (5 M) and diluted in α-MEM cell culture medium to obtain the range of tested concentrations. The final DMSO concentration did not exceed 0.2%, which is known to be nontoxic to *Leishmania* parasites [41,42]. For drug susceptibility assay, *Leishmania* growth curves were constructed by measuring absorbance at 600 nm [43]. The antileishmanial activity is expressed as IC_50_/72 h, which is the concentration that reduces cell growth by 50% compared to untreated control (relative growth). All experiments were done at least three times as independent experiments performed in triplicate.

#### 3.4.3. Cytotoxicity Assay against Peritoneal Macrophages

The concentration of studied compounds which is cytotoxic to 50% of the macrophages (CC_50_) was determined by 3-(4,5-dimethylthiazol-2-yl)-2,5 diphenyltetrazolium bromide (MTT) method according to Mosmann [44]. Macrophages were obtained by lavage of peritoneal cavity of mice with 10 mL cold RPMI without FBS. After washing, the cell suspension (4.0 × 10^6^/mL) was seeded (0.1 mL) in 96-well flat bottom plates. Macrophages were allowed to adhere for 2 h and non-adherent cells were removed by washing with RPMI. Then, the studied compounds were added to the wells at concentrations ranging from 1 to 20 μM and the cells were further cultured in RPMI-1640 supplemented with 10% FBS for 24 h at 37 °C in a humidified 5% CO_2_ atmosphere. Thereafter, the medium was replaced with fresh RPMI containing 0.5 mg/mL of MTT and the plates were incubated for additional 4 h. Supernatants were aspirated and the formazan crystals formed were dissolved in 100 μL of DMSO. After 15 min of incubation at room temperature, absorbance of solubilized MTT formazan product was spectrophotometrically measured at 570 nm.

### 3.5. Statistical Analysis

The IC_50_ and CC_50_ values were calculated by nonlinear regression using the software GraphPad Prism 5.0. The acceptable level of significance was 95% (*p* < 0.05).

### 3.6. n-Octanol/Water Partition Coefficient

Log P values were determined by the Shake-Flask method dissolving complexes **1** and **2** in octanol-saturated aqueous phosphate buffer (pH 6.8) to a concentration at which the solution was still unsaturated but close to saturation, 10 and 5 µmol·L^−1^, respectively. Then, three different partition system were prepared by adding these solutions to water-saturated octanol, in octanol/water volume ratios, 1:1, 1:2 and 2:1 in duplicate. The mixtures were vortexed for 10 min and then centrifuged for 5 min. The partition coefficients were determined using the absorbance of complexes **1** and **2**. Calculated dppz log P value was obtained from the program ALOGPS 2.143 available on line. The feasibility of ALOGPS 2.143 ability to predict partition coefficient was successfully demonstrated [45].

## 4. Conclusions

Two novel complexes of Sb(III) and Bi(III) with dppz were synthesized in reasonably good yields and characterized as [Sb(dppz)Cl_3_]∙H_2_O∙CH_3_OH and [Bi(dppz)Cl_3_]. These complexes were more effective than dppz alone and at least 77 and 2,400 times more active than potassium antimonyl tartrate against Sb(III)-sensitive and -resistant *Leishmania*, respectively. The lack of cross-resistance to the Sb(III)-dppz complex, together with the much lower activity of antimonyl tartrate, SbCl_3_ and BiCl_3_ strongly support the model that the metal is not active by itself but improves the activity of dppz through complexation. Complexation was found to decrease the lipophilicity of dppz, suggesting the role of this factor in the improved antileishmanial activity of the complexes. The present work successfully applied a novel approach, based on tests against metal-resistant mutants and their respective parental sensitive strains, to get insight into the role of the metal in the cytotoxicity of metal complexes.
